# Clinical care pathway program versus open-access system: a study on appropriateness, quality, and efficiency in the delivery of colonoscopy in the colorectal cancer

**DOI:** 10.1007/s11739-020-02565-z

**Published:** 2021-02-08

**Authors:** Giovanna Del Vecchio Blanco, Rami Dwairi, Mario Giannelli, Giampiero Palmieri, Vincenzo Formica, Ilaria Portarena, Enrico Grasso, Laura Di Iorio, Michela Benassi, Emilia Anna Giudice, Antonella Nardecchia, Piero Rossi, Mario Roselli, Giuseppe Sica, Giovanni Monteleone, Omero Alessandro Paoluzi

**Affiliations:** 1 Department of Systems Medicine, Gastroenterology Unit, University of Rome “Tor Vergata”, Rome, Italy; 2 Department of Internal Medicine, University of Mutah, Karak, Jordan; 3 Department of Biomedicine and Prevention, Anatomic Pathology Unit, University “Tor Vergata”, Rome, Italy; 4 Department of Oncohematology, Oncology Unit, University Tor Vergata, Rome, Italy; 5 Department of Diagnostic Imaging, Interventional Radiology and Radiotherapy, University “Tor Vergata”, Rome, Italy; 6 Department of Surgery, University Tor Vergata, Rome, Italy

**Keywords:** Clinical care pathway, Colonoscopy, Colorectal cancer, Open-access colonoscopy

## Abstract

Open-access colonoscopy (OAC), whereby the colonoscopy is performed without a prior office visit with a gastroenterologist, is affected by inappropriateness which leads to overprescription and reduced availability of the procedure in case of alarming symptoms. The clinical care pathway (CCP) is a healthcare management tool promoted by national health systems to organize work-up of various morbidities. Recently, we started a CCP dedicated to colorectal cancer (CRC), including a colonoscopy session for CRC diagnosis and prevention. We aimed to evaluate the appropriateness, the quality, and the efficiency in the delivery of colonoscopy with the open-access system and a CCP program in the CRC. Quality indicators for colonoscopy in subjects in the CCP were compared to referrals by general practitioners (OAC) or by non-gastroenterologist physicians (non-gastroenterologist physician colonoscopy, NGPC). Attendance rate to colonoscopy was greater in the CCP group and NGPC group than in the OAC group (99%, 99%, and 86%, respectively). Waiting time in the CCP group was shorter than in the OAC group (3.88 ± 2.27 vs. 32 ± 22.31 weeks, respectively). Appropriateness of colonoscopy prescription was better in the CCP group than in the OAC group (92 vs. 50%, respectively). OAC is affected by the lack of timeliness and low appropriateness of prescription. A CCP reduces the number of inappropriate colonoscopies, especially for post-polypectomy surveillance, and improves the delivery of colonoscopy in patients requiring a fast-track examination. The high rate of inappropriate OAC suggests that this modality of healthcare should be widely reviewed.

## Introduction

Colorectal cancer (CRC) is the second cause of cancer-related death in men and women in Western Countries [1, 2]. Prevention by fecal occult blood test (FOBT), sigmoidoscopy, and colonoscopy has been proven to reduce mortality and morbidity due to CRC [3, 4]. The diffuse awareness of the crucial role played by prevention and early diagnosis of CRC has led to an increasing prescription of colonoscopy by gastroenterologists and other specialists, but also by the general practitioner (GP). The GP plays a key role for subjects with worrisome symptoms or those who, although asymptomatic, wish to carry out an investigation for CRC prevention. The GP may prescribe an open-access colonoscopy (OAC) whereby the colonoscopy is performed without a prior office visit with a gastroenterologist [5]. OAC theoretically should facilitate access to an endoscopic procedure bypassing unnecessary visit, reducing the costs, and increase the number of subjects who undergo screening colonoscopy [6]. Indeed, over the years, OAC has been negatively affected by the inappropriateness of prescription [7, 8]. Post-polypectomy surveillance is a frequent indication of OAC, but a large proportion of colonoscopies are inappropriate in both selection of cases and timing of controls [9, 10]. Only 31% of the patients in whom an advanced adenoma was removed undergo a timely surveillance colonoscopy [11], while over 45% of patients without a high-risk adenoma removed at baseline colonoscopy receive too intense surveillance [12]. Several international guidelines [13–18] have been released to better define the indication, age for starting screening, and intervals of surveillance controls. Indeed, a deviation from guidelines up to 67% has been reported [12, 19, 20]. Therefore, many useless colonoscopies are performed with a significant increase of costs, risks of procedure-related complications, and consequently reduced availability of the procedure when it is urgent for alarm symptoms [12, 21, 22].

In the last few years, the clinical care pathway (CCP) programs have been promoted worldwide by the national health systems (NHS) to organize the diagnostic and therapeutic work-up of various morbidities. Based on guidelines and clinical practice, the purpose of every CCP is to increase the appropriateness of diagnostic procedures, shorten the waiting time, tailor the therapy, and reduce costs. In 2014, a multidisciplinary CCP aimed for prevention, diagnosis, and treatment of CRC was activated in the hospital of University Tor Vergata [23]. As a part of the CCP, an endoscopy session was organized to perform a fast-track colonoscopy in patients with a likelihood of cancer, but also for CRC prevention.

We aimed to evaluate the appropriateness, the quality, and the efficiency in the delivery of colonoscopy in a CCP program compared to OAC and colonoscopy prescribed by specialists other than a gastroenterologist in the CRC.

## Methods

### The clinical care pathway PDTA TCR

In 2014, a multi-specialist CCP dedicated to the prevention, diagnosis, treatment, and follow-up of CRC, termed as PDTA TCR [in Italian, Percorso Diagnostico Terapeutico Assistenziale (PDTA) per il Tumore Colo-Rettale (TCR)], was started at the Policlinico Tor Vergata, the hospital of the University Tor Vergata. The PDTA TCR CCP includes clinical visits, investigations, and treatments delivered by gastroenterologists, oncologists, radiotherapists, and surgeons. Among the activities of the CCP, a weekly colonoscopy session is included. Indications to colonoscopy of CCP (CCPC) are a likelihood of CRC, post-cancer resection follow-up, and a personal history of any cancer other than CRC (oncological screening). For the present study, from November 2015 to April 2017, CCPC has been also proposed for CRC prevention to patients undergoing a gastroenterological visit for conditions not related to CRC (i.e., dyspepsia, gastro-oesophageal reflux, peptic ulcer, Helicobacter pylori infection, gastritis, biliary disorders, and celiac disease) not reached by or who had not joined the national CRC screening program. During the visit, physicians operating in the CCP provide detailed information about the benefits of colonoscopy procedure, the importance of high-quality bowel preparation, the type of sedation, possible adverse events related to the procedures and medications, the need to respect appropriate intervals in the post-polypectomy surveillance to avoid unnecessary controls, and how to cancel the appointment. Candidates for colonoscopy receive a prescription for making the appointment through the NHS in a list not accessible to GP or other specialists.

### Study population and data collection

All subjects submitted to CCPC for CRC prevention, post-polypectomy surveillance, and a self-prescribed positive FOBT, with/without a CRC family history, were prospectively enrolled in the study. Subjects scheduled for a colonoscopy with the same indications, on the same day of the CCPC, prescribed by GP (OAC) or by a non-gastroenterologist physician (NGPC) working at the Tor Vergata University Hospital, were considered as controls. After the informed consent was given, all subjects were interviewed before colonoscopy. All demographic and clinical data have been recorded in an electronic database. Among items considered during the interview were: weight, height, body mass index (BMI), waiting time for a colonoscopy, CRC family history, personal history of any cancer other than CRC, diet, lifestyle (smoking habit and alcohol intake), medicine in active therapy, the result of FOBT, and findings of a previous colonoscopy, when performed. Patients underwent colonoscopy after carried out a split-dose high or small volume bowel preparation. Colonoscopy was performed up to the cecum under deep (propofol) or conscious sedation (fentanyl and/or midazolam). Conditions requiring the immediate interruption of the examination, including inadequate bowel cleansing, stenosis due to cancer or diverticular disease, surgical adherence, adverse events to medications, or complications during the exam, were recorded. At the end of colonoscopy, the type of sedation, lesions found, and grade of bowel preparation were recorded in the study database. Bowel preparation was scored according to the Boston bowel preparation scale (BBPS) [24, 25]. The lesions were classified as non-dysplastic polyps, adenomas, advanced adenomas (presence of at least one of the following features: size > 10 mm, high-grade dysplasia, and villous morphology), and cancer.

### Evaluation of data and statistical analysis

Quality indicators for colonoscopy previously defined [14, 26] were compared in the three study groups: attendance rate, the waiting time, appropriateness of prescription, quality of bowel preparation, cecal intubation rate, and adenoma detection rate. The attendance rate to colonoscopy was quantified by matching subjects who turned up to perform the colonoscopy with respect to those who were included on the appointment lists. The waiting time for a colonoscopy was calculated as the number of weeks between the date of reservation and the day of colonoscopy. Appropriateness of colonoscopy prescription was defined according to the respect of the criteria set in the guidelines for screening (start and stop age) and post-polypectomy surveillance (interval of controls according to the histology and number of lesions at baseline colonoscopy) [14]. Bowel preparation was evaluated following the European guidelines [26] and deemed as adequate when the BBPS score was  ≥ 6. Both cecal intubation rate and adenoma detection rate (ADR) were calculated.

Statistical analysis of data was made using IBM Corp. released in 2012. IBM SPSS Statistics for Windows, Version 21.0. (IBM Corp., Armonk, NY, USA). Data were evaluated by univariate analysis, summarized with mean ± standard deviation if related to continuous variables and with percentages when referring to categorical ones. Statistical significance between groups was determined using the χ^2^ test and Student’s *t* test. A *P* value of < 0.05 was considered statistically significant.

## Results

### Study population

**Fig. 1:**
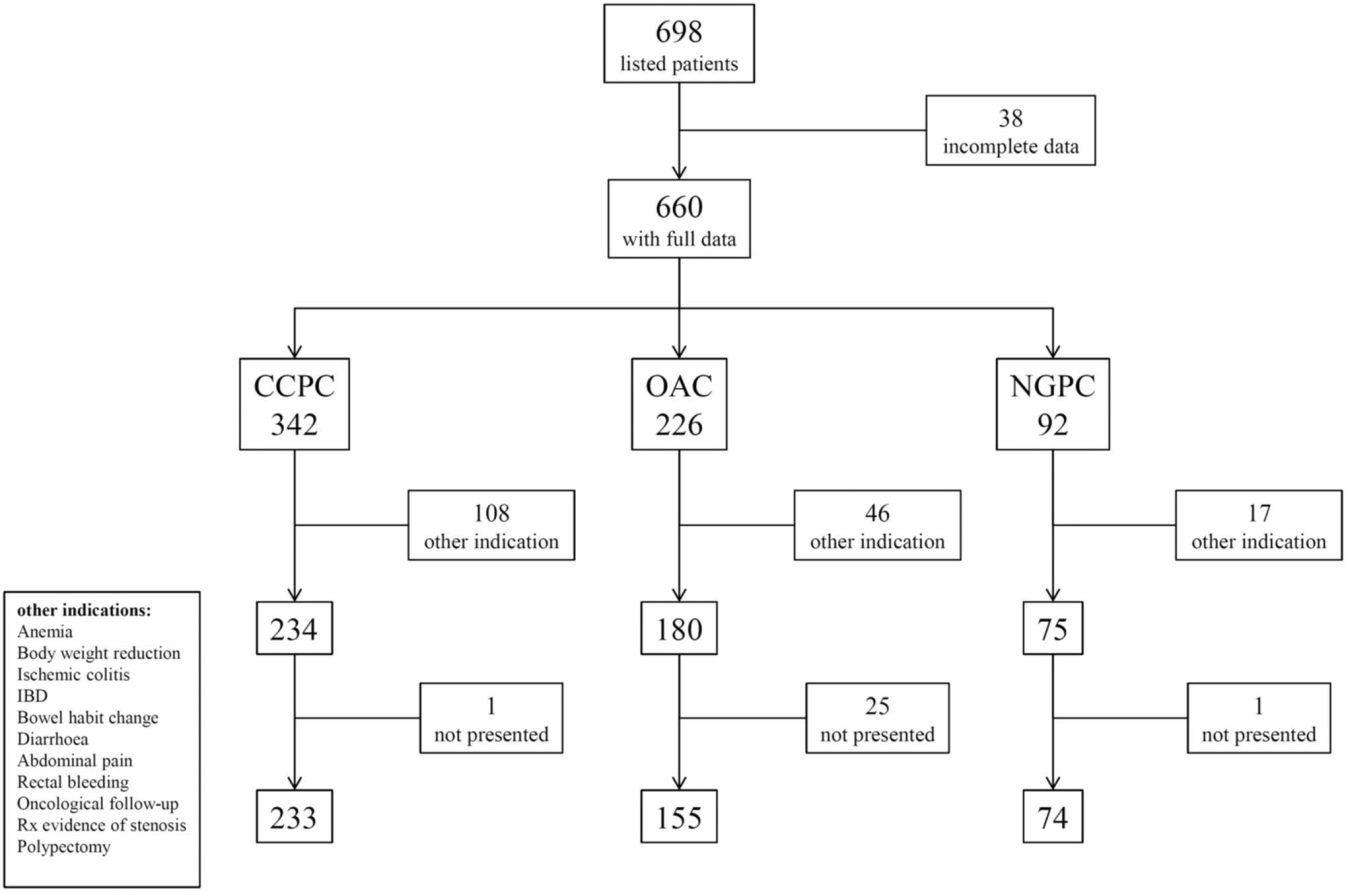
Study population

A total of 698 subjects were recorded in the database, of whom 38 with incomplete data and 171 undergone colonoscopy for indications other than those considered in the study were excluded from the analysis. Therefore, 489 subjects made up the study population, 234 in the CCP group, 180 in the OAC group, and 75 in the NGPC group. Out of 489 subjects, 462 (94.5%) underwent colonoscopy, 233 in the CCP group, 155 in the OAC group, and 74 in the NGPC group (Fig. 1). Clinical features of subjects who attended colonoscopy are shown in Table 1. Of the 462 subjects, 174 (38%) had  ≥ 1 first-degree relative (FDR) with CRC, while the remaining 288 subjects (62%) did not report a CRC family history. The proportion of subjects with a CRC family history in the NGPC group was higher than in the CCP and OAC groups (*P* < 0.01). No difference was found comparing subjects according to age, gender, smoking habits, and BMI.

**Table 1:** Characteristics of the study population

Characteristics	Overall, *n*/*N* (%)	CCPC, *n*/*N* (%)	OAC, *n*/*N* (%)	NGPC, *n*/*N* (%)	*P*
Subjects enrolled	489	234	180	75	
Subjects who attended colonoscopy	462/489 (94%)	233/234 (99%)	155/180 (86%)	74/75 (99%)	< 0.01
Age (mean ± SD, years)	62 ± 11	60 ± 11	62 ± 12	60 ± 12	0.21
Gender (males/females)	224/238	108/125	80/75	36/38	0.59
CRC family history					
Yes	174/462 (38)	78/233 (33)	52/155 (44)	44/74 (59)	< 0.01
No	288/462 (62)	155/233 (67)	103/155 (66)	30/74 (41)	
Smoking					
Never	321/462 (69)	173/233 (74)	103/155 (66)	45/74 (61)	0.08
Former	67/462 (15)	26/233 (11)	29/155 (19)	12/74 (16)	
Current	74/462 (16)	34/233 (15)	23/155 (15)	17/74 (23)	
BMI					
< 25	220/462 (47)	106/233 (45)	71/155 (46)	43/74 (58)	0.15
25–29.9	164/462 (35)	80/233 (34)	61/155 (39)	23/74 (31)	
≥ 30	78/462 (18)	47/233 (20)	23/155 (15)	8/74 (11)	

*CPC* clinical care pathway colonoscopy, *OAC* open-access colonoscopy, *NGPC* non-gastroenterologist physician colonoscopy

### Colonoscopy indicators

#### Attendance to a colonoscopy

Out of 489 subjects, 27 (5.5%), one in the CCP group, 25 in the OAC group, and one in the NGPC group, did not present to perform a colonoscopy. Therefore, the attendance rate to colonoscopy was 99% in the CCP and NGPC groups versus 86% in the OAC group (*P* < 0.01).

#### Waiting time for colonoscopy

**Fig. 2:**
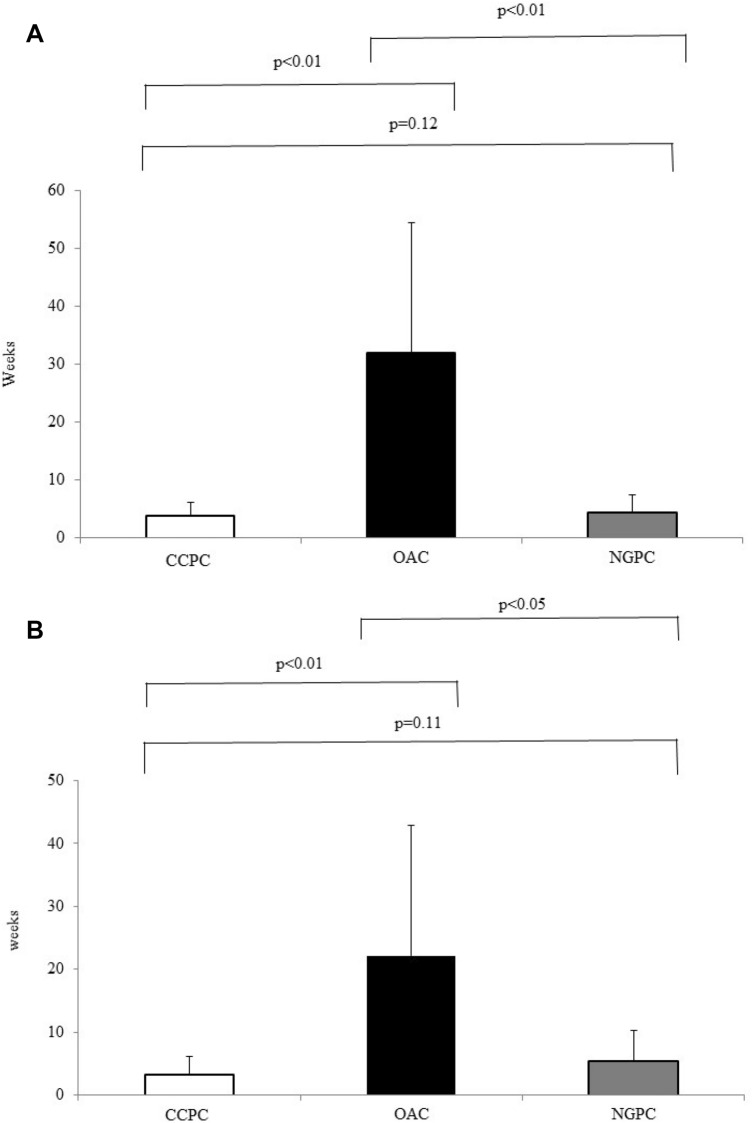
**a** Overall mean waiting time for colonoscopy in the OAC group was significantly longer than in the CCPC group and NGPC group (32 ± 22.31 vs. 3.88 ± 2.27 and 4.38 ± 2.95 weeks, respectively; *P* < 0.01). **b** Mean waiting time for colonoscopy in subjects having a positive FOBT in the OAC group was significantly longer than in the CCPC group and NGPC group (22.15 ± 20.65 vs. 3.25 ± 2.81 and 5.33 ± 4.89 weeks*,* respectively; *P* < 0.05). No difference was found comparing the waiting time for colonoscopy in the CCP and NGPC groups. CCPC: clinical care pathway colonoscopy; OAC: open-access colonoscopy; NGPC: non-gastroenterologist physician colonoscopy

Overall, the waiting time in the OAC group was significantly longer than in the CCP group and NGPC group (32 ± 22.31 vs. 3.88 ± 2.27 and 4.38 ± 2.95 weeks, respectively; *P* < 0.01). Comparing FOBT positive subjects, waiting time in the OAC group was longer than in the CCP group and NGPC group (22.15 ± 20.65 vs. 3.25 ± 2.81 and 5.33 ± 4.89 weeks, respectively; *P* < 0.05) (Fig. 2), while was similar in the CCP group and NGPC group.

#### Sedation

Colonoscopy was performed under conscious sedation in 404 subjects (88%) and deep sedation in 20 (4%), while in 38 (8%), no sedation was administered by patient choice. When asked the reason why sedation was refused, 19 answered they had already undergone a colonoscopy without sedation. In contrast, among the ones remaining 19, performing colonoscopy for the first time, 14 declared to prefer maintaining control of consciousness and 5 to be afraid of adverse events.

#### Indication to colonoscopy

Indication to colonoscopy was CRC prevention in 336 subjects (73%), post-polypectomy surveillance in 77 subjects (17%), and positive FOBT in 49 subjects (10%). When comparing indications to colonoscopy among the three groups, CRC prevention was more frequent in the CCP group and NGPC group, post-polypectomy surveillance in the OAC group, and positive FOBT in the NGPC group (*P* < 0.01).

#### Appropriateness of colonoscopy prescription

**Fig. 3:**
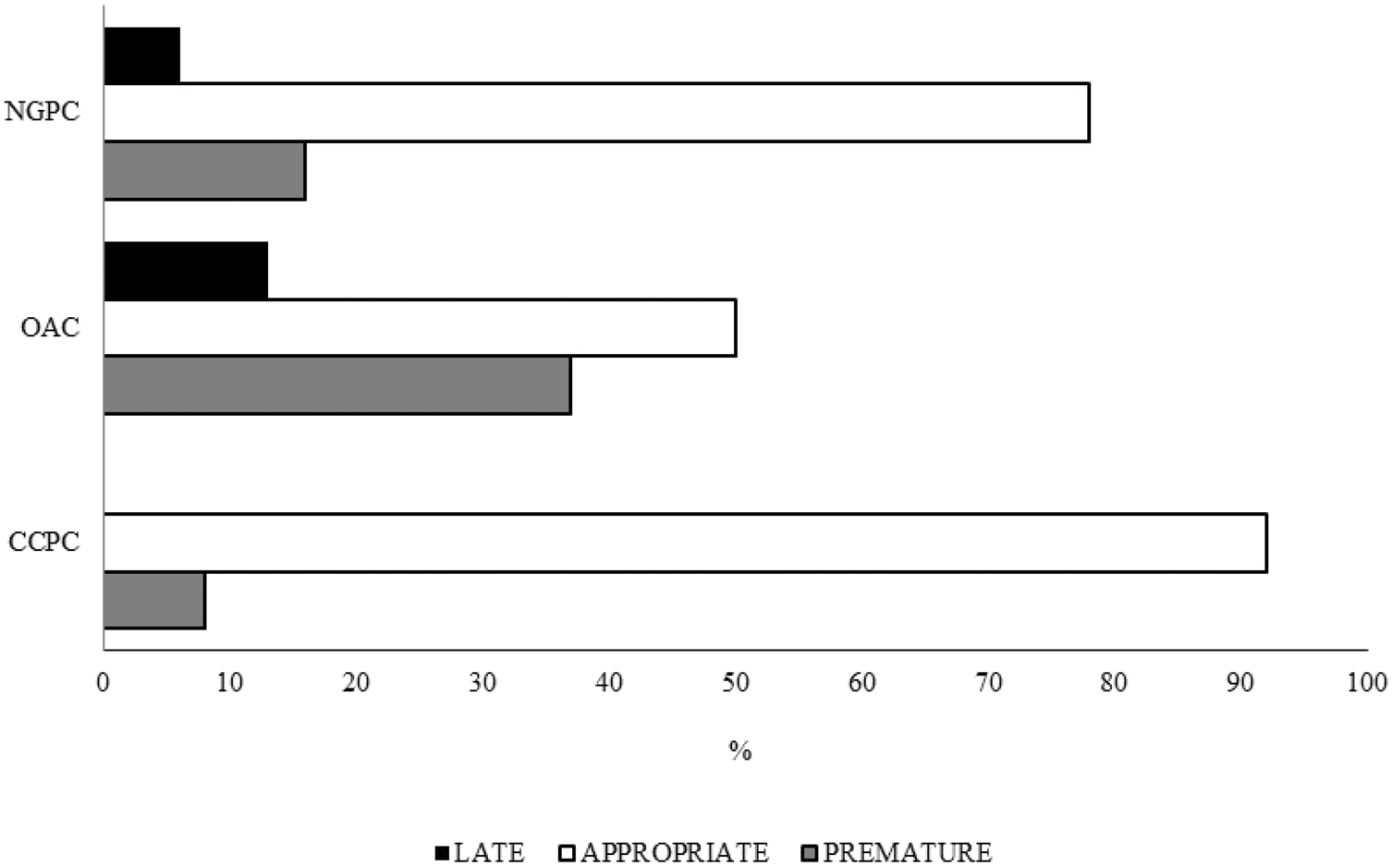
Appropriateness of colonoscopy timing in the three subjects groups undergone colonoscopy for CRC prevention or post-polypectomy surveillance: CCPC = 92%, OAC = 50%, NGPC = 78%; *P* < 0.001. CCPC: clinical care pathway colonoscopy; OAC: open-access colonoscopy; NGPC: non-gastroenterologist physician colonoscopy

Out of 462 colonoscopy prescriptions, 350 (76%) were deemed as appropriate. In the CCP group, 18/233 colonoscopies (8%) were inappropriate as earlier than recommended. In the OAC group, 78/155 colonoscopies (50%) were inappropriate, 58 early and 20 late examinations. In the NGPC group, 16/74 colonoscopies (22%) were inappropriate, 12 early and 4 late examinations. Overall, the appropriateness of colonoscopy in the CCP group resulted significantly higher than in the OAC and NGPC groups (92 vs. 50% and 78%, respectively; *P* < 0.001) (Fig. 3).

#### Adequacy of examination

Out of the 462 colonoscopies, 414 were complete, with an overall cecal intubation rate of 89.6%. No difference was found in comparing the cecal intubation rate in the three groups (CCP 89.2%, OAC 89.6%, NGPC 90%; *P* = 0.83). The reasons for incomplete colonoscopy in 48 out of 462 subjects (11.4%) are shown in Table 2. In the majority of the cases, colonoscopy was discontinued due to poor bowel preparation (25/48 subjects, 52%) or impassable strictures (14/48 subjects, 29%). Colonoscopy was interrupted due to intolerance in eight subjects (17%), in seven of whom the exam was performed under conscious sedation and in one with no sedation. In one subject, colonoscopy was brought to a stop due to the occurrence of arrhythmia. Excluding colonoscopies in which premature interruption of the examination was due to impassable strictures and arrhythmia, the cecal intubation rate was 93%.

**Table 2:** Reasons of incomplete colonoscopy

Conditions	Overall, *n*/*N* (%)	CCPC, *n*/*N* (%)	OAC, *n*/*N* (%)	NGPC, *n*/*N* (%)	*P*
Total number of colonoscopies	462	233	155	74	
Incomplete colonoscopies	48/462 (10.4)	25/233 (10.7)	16/155 (10.3)	7/74 (9.4)	0.95
Poor bowel preparation	25/48 (52)	11/25 (44)	9/16 (56)	5/7 (71)	0.68
Impassable strictures	14/48 (29)	8/25 (32)	6/16 (37)	–	0.17
Cancer	5/48 (10)	3/25 (12)	2/16 (12.5)	–	
Diverticular disease	9/48 (19)	5/25 (20)	4/16 (24.5)	–	
Intolerance	8/48 (17)	5/25 (20)	1/16 (7)	2/7 (29)	0.33
Arrhythmia	1/48 (2)	1/25 (4)	–		0.62

*CPC* clinical care pathway colonoscopy, *OAC* open-access colonoscopy, *NGPC* non-gastroenterologist physician colonoscopy

In 414 full colonoscopies, the mean BBPS score was 7.14. No statistically significant difference was found comparing the mean BBPS score in the three groups (CCPC 7.08 ± 1.34; OAC 7.18 ± 1.72; NGPC 7.22 ± 0.79; *P* = 0.72). Bowel preparation was similar in both male and female subjects.

Colonoscopy findings are shown in Table 3. A total of 198 polyps were removed during 146 out of 462 colonoscopies (32%), 141 were non-advanced adenoma, and 12 advanced adenoma, while the remaining 45 polyps were non-dysplastic. No lesion was detected in the remaining 316 colonoscopies. Overall, ADR was 32%. No difference among ADR in the three study groups was found (32% in the CCP group, 32% in the OAC group, and 30% in the NGPC group, *P* = 0.79). Adenocarcinoma was diagnosed in nine subjects (1.95%) of whom four were FOBT-positive, causing an impassable stricture in five cases. No difference was found comparing the incidence of lesions in patients with different CRC family history.

**Table 3:** Endoscopic findings in 462 subjects submitted to colonoscopy for screening, post-polypectomy surveillance or positive FOBT

Characteristics	Overall,* n*/*N* (%)	CCPC, *n*/*N* (%)	OAC, *n*/*N* (%)	NGPC, *n*/*N* (%)
Total number of colonoscopies	462	233	155	74
Number of polypectomies	198/462 (43)	85/233 (36)	72/155 (46)	41/74 (55)
Adenocarcinoma	9/462 (1.95)	5/233 (2)	4/155 (2.6)	–
Advanced adenoma	12/198 (2.5)	5/233 (2)	6/155 (3.8)	1/74 (1)
Non-advanced adenoma	141/198 (30.5)	71/233 (30.5)	44/155 (28.6)	26/74 (35)
Non-dysplastic polyps	45/198 (10)	9/233 (4)	22/155 (14)	14/74 (19)

*CPC* clinical care pathway colonoscopy, *OAC* open-access colonoscopy, *NGPC* non-gastroenterologist physician colonoscopy

#### Complications to a colonoscopy

No relevant complications occurred in the study population. However, one case of arrhythmia (atrial fibrillation) occurred once reaching the transverse colon in a 58-aged male, undergoing screening colonoscopy for the first time, which was promptly reverted by electrical cardioversion.

## Discussion

The present study shows that the OAC is affected by the lack of timeliness which is at least partly due to the reduced appropriateness of prescription. The delivery of colonoscopy in a short time is a crucial point, especially in patients with alarming symptoms or with a positive FOBT. Recent evidence [27, 28] highlighted that every month more until colonoscopy is associated with an increased risk of a CRC in advanced stage and mortality risk in subjects with a positive FOBT. Thus, a fast-track colonoscopy, within 1 month, is mandatory in patients with a likelihood of CRC and FOBT-positive subjects [29, 30]. An analysis carried out by University Tor Vergata, in collaboration with the Italian GP association, estimated a mean national waiting time of 96 days and 175 days in our district (Regione Lazio) for an OAC in 2017 [31]. Waiting times for colonoscopy are long, although shorter than in Italy, also in other countries, 94 days in Canada, 76 days in Kent and Midways, and 53 days in Australia [32–34]. In the present study, OAC had a mean waiting time of 32 weeks. The CCP here evaluated allowed reducing the waiting time for a colonoscopy considerably. Such a finding is not surprising, as the access to appointment list was restricted to personnel included in the CCP. What is clinically relevant is that all patients, especially those with a positive self-prescribed FOBT, included in the CCP group had the opportunity to undergo colonoscopy within a mean time of 3 weeks. Several factors may influence waiting time for an OAC. First, the high rate of no-shows or late cancellations, in the present study accounting for 14% in the OAC group. This is in agreement with a recent study in which OAC was burdened by 13.5% of no-show [8]. Overprescription of colonoscopy related to non-adherence to international guidelines is another reason for long waiting time. Deviation from guidelines has been reported in different countries [10, 33, 35]. The inappropriateness of OAC mainly concerns post-polypectomy surveillance and has been reported with rates ranging from 25 to 50% [7, 12, 19, 20, 36, 37]. In a recent Italian survey [19], two-thirds out of  ~ 50,000 colonoscopies delivered within the NHS from different areas of Italy were inappropriate. In our investigation, inappropriateness in the CCP group was very limited (8%), while half of OACs were inappropriate. These findings seem to suggest that (1) improving adherence to guidelines may reduce waiting time for an OAC, in agreement with other evidence [38]; (2) gastroenterologists are more familiar with the guidelines than GP. Awareness of post-polypectomy surveillance guidelines is not the only reason for the inappropriate timing of colonoscopy [39]. Other possible factors are new clinical signs and symptoms, inadequate bowel preparation, concern for missed synchronous or metachronous lesions, and associated medico-legal consequences, referring physician or patient insistence [38–40].

The attendance to a colonoscopy in the CCP group was greater than OAC. This finding, probably more related to the delivery of examination in a shorter time than the program per se, reflects the main limitation of OAC, the lack of timeliness which induces patients to look for other colonoscopy opportunities.

The performance of OAC and non-OAC was similar in the present study. The cecal intubation rate, quality of bowel preparation, and the outcome of endoscopy were similar in the three groups, with comparable ADR and malignancy detection rate. Our findings are consistent with prior studies reporting a similar diagnostic yield of OAC and non-OAC [7, 8, 41–43]. The lack of difference in the outcome in the three groups induces to speculate that the CCP program may be more useful, especially in symptomatic subjects who need a colonoscopy in a short time.

Overall, a CCP program seems to be useful to rationalize the prescription and the delivery of colonoscopy as it increases the attendance rate, improves the appropriateness of the prescription, and shortens the waiting time for a colonoscopy. The access to such a CCP is through a visit with a gastroenterologist or one of the other specialists involved in the program. The possible increase in the request for office visits may be compensated for by reducing inappropriate colonoscopies. Many countries in the western world have CCP in which the GP indicates the level of priority in prescribing an investigation. In contrast, Italy has not yet widely implemented the CCP which, in cooperation with GP, could make a strong contribution to the rationalization of resources. Further studies are necessary to define this issue.

Our study has several limitations. First, criteria to access colonoscopy are quite heterogeneous. Indeed, these criteria represent the indication of colonoscopy in the clinical practice and are conditions associated with the CRC for which our CCP called PDTA TCR was conceived. We have restricted this heterogeneity by including only colonoscopies for CRC prevention and the positivity of FOBT in the analysis of data. Second, our findings referring to a monocentric investigation in a limited period (< 2 years). Nevertheless, our data are in keeping with those emerging from a previous survey-based national Italian study [19]. Third, the majority of the subjects in the study were 50–75 years of age, but had not entered an organized screening program. Despite this should be considered as a bias of the study, it is a fact that a proportion of subjects in the real life, especially in the central and southern Italian regions, do not uptake the screening proposal. These subjects, reluctant or not well informed about the benefits of screening, are probably those who need specialist counseling to raise awareness and confidence in the organized screening which represents the best CRC prevention strategy.

In conclusion, the present study demonstrates that OAC is affected by the lack of timeliness and low appropriateness of prescription. A CCP reduces the number of inappropriate colonoscopies, especially for post-polypectomy surveillance, and improves the delivery of colonoscopy in patients requiring a fast-track examination. The high rate of inappropriate OAC in the present study suggests that this modality of healthcare should be widely reviewed. Greater cooperation between gastroenterologists and GPs could improve the awareness of guidelines and increase the appropriateness of colonoscopy.
